# Case report of the first use of a hydrogel rectal spacer for prostate cancer reirradiation via LDR brachytherapy: applications and technical notes

**DOI:** 10.3389/fonc.2025.1494304

**Published:** 2025-01-30

**Authors:** Amina Lazrek, Sebastiano Finocchi Ghersi, Adeline Petre, Sarah Houabes, Anne-Agathe Serre, Frederic Gassa, Magali Sandt, Cecile Laude, Camille Roukoz, Salvatore Cozzi

**Affiliations:** ^1^ Radiation Oncology Unit, International University Hospital Cheikh Zaid, Rabat, Morocco; ^2^ Radiation Oncology Unit, Azienda USL-IRCCS di Reggio Emilia, Reggio Emilia, Italy; ^3^ Radiation Oncology Department, Centre Leon Berard, Lyon, France; ^4^ Radiation Oncology Unit, Portes de Provence Hospital Groupe, Montelimar, France

**Keywords:** rectal spacer, hydrogel spacer, prostate reirradiation, prostate cancer, brachytherapy

## Abstract

**Introduction:**

Prostate cancer remains a prevalent malignancy among men, often necessitating innovative therapeutic strategies for effective management of recurrent cases. This article examines the critical role of a biodegradable hydrogel spacer, which creates a temporary interspace between the prostate and the rectum, thus reducing radiation exposure to healthy tissues.

**Case description:**

We present a case of a man with a history of intermediate-risk prostate adenocarcinoma initially treated with external beam radiotherapy in 2015. Despite initial remission, the patient experienced a rise in prostate-specific antigen (PSA) levels indicative of local recurrence in 2022. Salvage treatment with iodine-125 brachytherapy, preceded by the placement of a rectal spacer in January 2024, resulted in a significant reduction of PSA levels. The patient remains asymptomatic with no urinary or gastrointestinal complications 6 months after the salvage treatment.

**Discussion:**

This case illustrates the complexities in managing recurrent prostate cancer and the evolving role of reirradiation strategies. Salvage iodine-125 brachytherapy with the placement of a rectal spacer provided precise radiation delivery while minimizing rectal toxicities. The significant biochemical response observed underscores the efficacy of this approach in controlling disease progression. The rectal spacer enhances treatment safety by reducing radiation exposure to adjacent tissues, highlighting its importance in reirradiation protocols. This case contributes to the growing evidence supporting the rectal spacer’s role in enhancing the safety and efficacy of salvage brachytherapy for recurrent prostate cancer.

**Conclusions:**

Our experience advocates for the integration of a hydrogel rectal spacer as a valuable tool in prostate cancer reirradiation protocols, offering a strategic approach to optimize treatment safety by minimizing rectal toxicity.

## 1 Introduction

Prostate cancer (PC) persists as one of the most prevalent malignancies among the male population. Primary definitive radiotherapy (RT) with or without concurrent and adjuvant androgen deprivation therapy (ADT) represents a milestone in the treatment of non-metastatic, hormone-sensitive PC with curative intent. Despite that, 30% to 47% of patients experience biochemical failure and clinical relapse (1–3).

Intraprostatic recurrence is still a challenge, and the development of innovative therapeutic strategies to manage intraprostatic recurrences is required. Within this context, as an alternative to ADT, reirradiation has emerged as a complex but promising technique, offering a viable therapeutic option for patients experiencing recurrence following primary conservative treatment (4).

Notably, among the advancements within this therapeutic domain, brachytherapy has demonstrated considerable potential due to its precision and efficacy in delivering high radiation doses directly to the tumor.

However, the process of reirradiation inherently carries substantial risks to adjacent healthy tissues, particularly to critical anatomical structures such as the rectum (5).

It is in this critical juncture that rectal spacer placement adopts a pivotal role. The rectal spacer consists of a biodegradable hydrogel that is meticulously engineered to create a temporary interspace between the prostate and the rectum, thereby significantly lowering radiation exposure to the rectal wall.

By effectively reducing the radiation dose received to the rectum, the spacer substantially enhances the safety profile of PC reirradiation (6). In 2017, a phase III trial published by Hamstra et al. showed statistically significant difference of the bowel quality of life (QOL) and reported acute and late toxicities when a hydrogel spacer was used before RT (7).

This article deals with the role of rectal spacer in the context of PC reirradiation via iodine-125 brachytherapy, underscoring its importance in minimizing rectal adverse effects. Through a comprehensive analysis, this manuscript explores the clinical benefits, procedural considerations, and potential implications of integrating hydrogel rectal spacer technology into reirradiation protocols for PC recurrence.

## 2 Case description

This is the case of a man aged between 70 and 75 years old, with a medical history significant for hypertension, hyperuricemia, and a stage 2 Hodgkin’s lymphoma diagnosed in 2021, which had a favorable prognosis.

In 2015, a favorable intermediate-risk prostate adenocarcinoma following the National Comprehensive Cancer Network (NCCN) risk stratification [American Joint Committee on Cancer (AJCC) 8th edition: cT2cN0M0; ISUP 2; with 10% grade IV] was discovered. The patient underwent exclusive external beam radiotherapy (EBRT), delivering a total dose of 80 Gy to the prostate gland and 46 Gy to the seminal vesicles in conventional fractionation (2 Gy per fraction in 40 fractions), without pelvic irradiation or concurrent ADT. The nadir prostate-specific antigen (PSA) was 0.59 ng/mL achieved in 2020.

**Figure 1:**
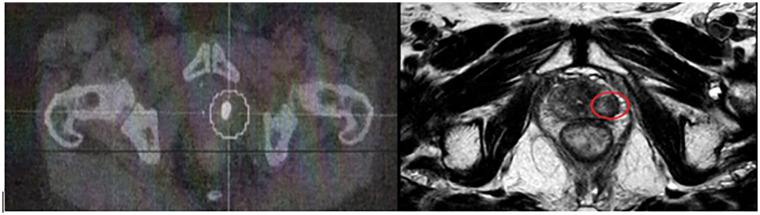
Left image: Choline PET scan: intense hypermetabolic activity in the posterior-superior part of the left prostatic lobe. Right image: T2 w image showing a 13-mm lesion (red circle) in the posterolateral peripheral zone.

Regular PSA monitoring revealed a progressive increase in values, and biochemical recurrence was confirmed when the PSA value reached the PSA nadir + 2 ng/mL. Indeed, in 2021, the PSA value was 2.55 ng/mL, so investigations followed. In July 2021, a PIRADS 4 lesion in the left posterolateral peripheral zone with a regular appearance of the capsule was identified using magnetic resonance imaging (MRI). Subsequently, a choline C-11 PET scan showed intense focal fixation at the left prostate gland without any suspicious regional node or distant metastases other than an abnormal activity on several cervical lymph nodes. A biopsy on these lymph nodes was done, and a Hodgkin lymphoma was diagnosed and treated by chemotherapy followed by cervical RT that ended in February 2022. At that time, local treatment on the prostate was not considered. A PET scan done by the end of 2022 showed a complete remission of the cervical lymph nodes and a metabolic progression of the left prostatic lesion. A new MRI was then done in February of 2023 and showed a local progression of the left prostatic lesion with a high suspicion of capsular invasion. The PSA level at that time was 4.89 ng/mL. Randomized prostate biopsies performed in June 2023 revealed an ISUP grade 5 adenocarcinoma in three out of six biopsies on the left side on the base and the apex. The six biopsies performed on the right lobe were all negative. A prostate MRI in February 2023 identified a 13-mm lesion PIRADS 4 in the left posterolateral peripheral zone extending from the base to the apex, with an intact capsule (**Figure 1**). Due the complete remission of his Hodgkin lymphoma, a local treatment for the prostate was decided and scheduled for January 2024. The pretreatment PSA was 7.08 ng/mL with a PSA doubling time of 19 months. Salvage prostate brachytherapy with iodine-125 was performed in January 2024. Concomitant ADT was not delivered. Low-dose-rate (LDR) brachytherapy was chosen over stereotactic body radiation therapy (SBRT) due to the strongest evidence available in the literature concerning the efficacy of LDR brachytherapy in the treatment recurrent PC. Partial gland irradiation was chosen based on the concordant findings of the biopsies, MRI, and PET scan, all of which indicated a recurrence on the left side with no evidence of recurrence on the right.

### 2.1 Hydrogel rectal spacer placement and brachytherapy

During the entire procedure, the patient was under general anesthesia in a gynecological position. The first step was the positioning of the hydrogel rectal spacer according to the procedure described by Montoya et al. (6). The rectal spacer procedure involves the application, via a transperineal approach, of an injectable and absorbable hydrogel designed to establish a transient separation between the rectum and the prostate.

**Figure 2:**
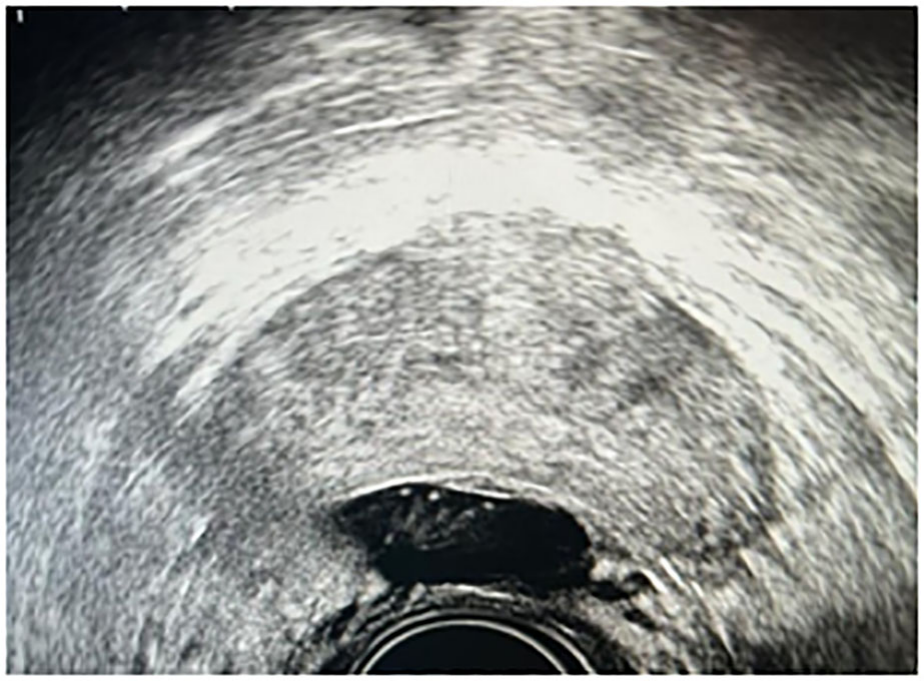
Ultrasound image showing the right placement of hydrogel between the prostate and the rectal wall.

An 18 G needle, connected to a syringe containing injectable physiological saline, is utilized under the guidance of real-time transrectal ultrasound (TRUS). The needle is advanced through the rectourethral muscle toward the peri-rectal adipose tissue, typically targeting the central region of the prostate, with careful axial verification to ensure its precise placement. Incremental injection of physiological saline expands the peri-rectal space, while systematic aspiration ensures avoidance of intravascular administration. Following this, the needle is maintained *in situ* during syringe removal, after which the hydrogel applicator is meticulously attached. A slow and uninterrupted injection of 10 mL of hydrogel effectively fills the peri-rectal space (**Figure 2**).

In a second time, real-time TRUS image-guided permanent iodine-125 source placement through transperineally implanted catheters was performed. Visualization of the urethra is accomplished by placing a Foley catheter. Needles are typically placed under TRUS image guidance through holes in a physical template that is mounted against the patient’s perineum. The approximate locations of the templated holes are superimposed on the ultrasound monitor image in order to facilitate real-time guidance. LDR brachytherapy sources can be preloaded into the needles for subsequent deposition or inserted through hollow needles in the prostate. In consideration of the extent of the disease, reirradiation of only the left prostatic lobe was performed by inserting 14 needles onto a target volume of 11 cc for a total dose of 145 Gy.

**Figure 3:**
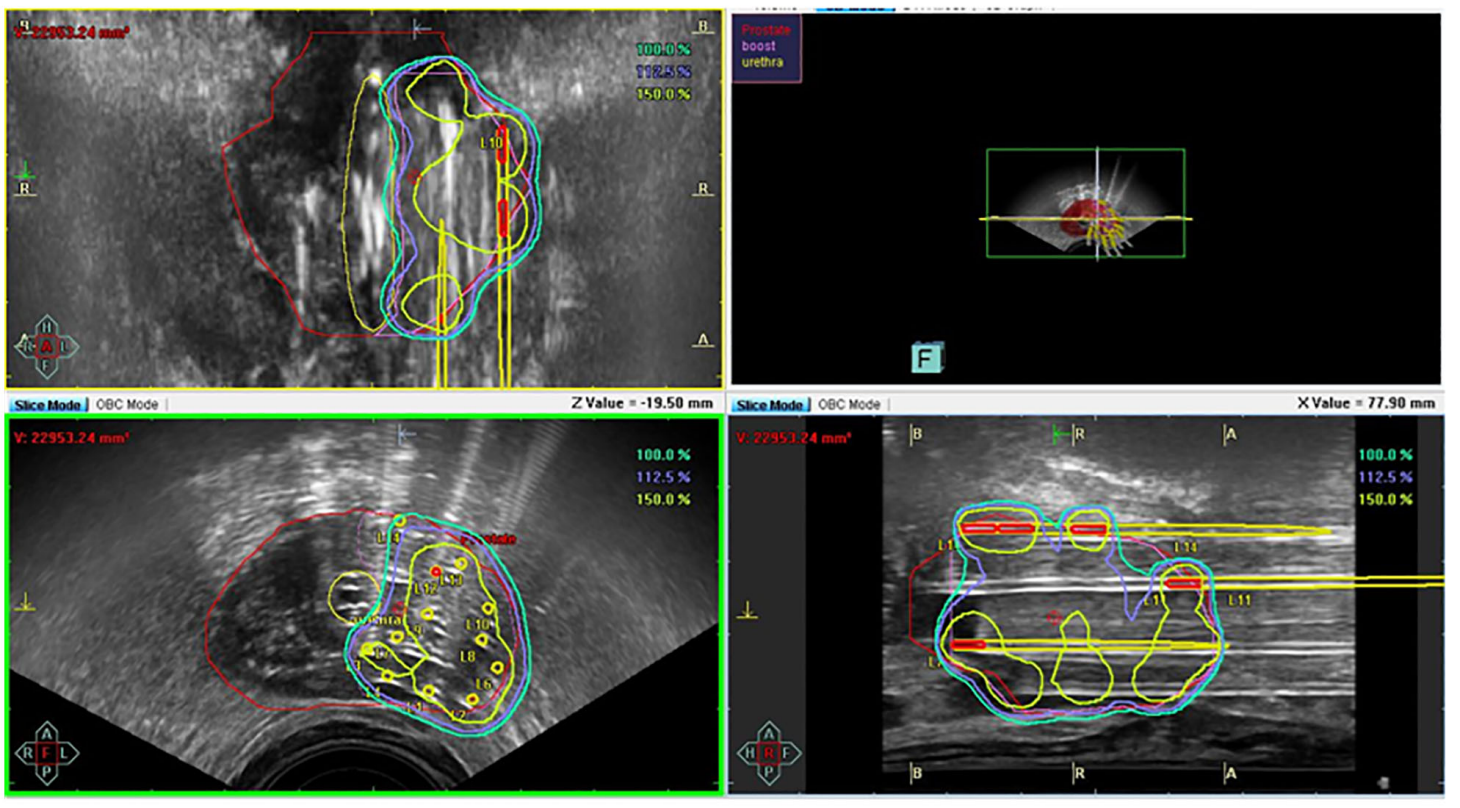
Brachytherapy implant for reirradiation and dose–volume histogram.

Concerning the dose–volume histogram (DVH) parameters, for the rectum, D2 cc was 65 Gy and D0.1cc was 78% of the prescribed dose. The average distance between the rectum and the prostate was approximately 45 mm (**Figure 3**).

### 2.2 Follow-up

After 6 months of follow-up, the patient is in good general condition with the WHO performance status equal to zero. He did not develop any urinary or gastrointestinal toxicities after the procedure and up to 6 months of follow-up. One month after the procedure, the PSA dropped significantly to a value of 2.41 ng/mL compared with the pre-intervention value of 7.08 ng/mL, demonstrating a good biochemical response.

## 3 Discussion

RT plays a crucial role in the management of PC with curative intent as well as in the salvage setting. Emerging data have established RT as an also useful therapeutic option for oligometastatic and oligorecurrent/oligoprogressive disease, for rare histologies, or in combination with new drugs available for hormone-sensitive and castration-resistant PC (8–10). In this setting, ADT represents the current approach, with a well-known but time-limited benefit and some unavoidable consequences on the patient’s QOL. These patients could still benefit from a local treatment with the aim of achieving a local control of the disease (11, 12). Salvage prostatectomy may offer a chance of cure. In recent years, advances in radiation planning and delivery techniques have improved treatment accuracy and given rise to the adoption of ultrahypofractionated radiation schedules in the form of SBRT in different oncological settings, with an acceptable toxicity profile (13–19). Indeed, Cuccia et al. conducted a systematic review on PC reirradiation, reporting low rates of acute and chronic grade 3 toxicities (20). They also reported the 1-, 2-, and 3-year biochemical relapse-free survival (BRFS) rates following reirradiation for PC to range between 79% and 85%, 40% and 73%, and 55% and 69%, respectively, reflecting promising intermediate-term outcomes in appropriately selected patients. Distant progression-free survival rates were reported only in few SBRT studies, ranging between 53% and 54% at 2 years (20). Their findings underscore the safety and feasibility of modern reirradiation techniques, making them a viable option for carefully selected patients. Following the uro-oncological Study Group of the Italian Association of Radiotherapy and Clinical Oncology (AIRO), ADT was not added in our patient. Indeed, most AIRO members agreed that prescription of concomitant ADT should be based on a case-by-case analysis (21).

The critical issue in reirradiation is the tolerability of previously irradiated organs at risk (OARs) that could preclude a dose with curative intent and consequent sequelae such as anastomotic stricture, urinary incontinence, and rectal injury. In this scenario, various RT techniques have been used, and among these, brachytherapy is the technique with the longest follow-up and allows a higher sparing of OAR, with their sharper gradient of dose while maintaining ablative dose, with promising results (22). LDR brachytherapy requires a skilled brachytherapy physician working in concert with physicists, dosimetrists, and others to perform a technique that is safe, reproducible, and robust to inherent clinical uncertainties.

Several studies have reported the use of interstitial brachytherapy in the reirradiation setting, showing the best therapeutic window with the highest biochemical control rate and the lowest prevalence of urinary incontinence and obstruction compared with other local therapies (i.e., salvage prostatectomy or high-intensity focused ultrasound) (23–49). The most significant acute and late toxicities occurred in the genitourinary domain. Regarding rectum toxicity, Dipasquale et al. demonstrated that the volume of rectum that received more than 70 Gy at primary RT course was a strong predictor of late rectal toxicities (50). Moreover, when summing the primary and reirradiation doses delivered to 1 cc of the rectum, a threshold dose of 130 Gy (a/b of 3 Gy) was found to be significantly related to the risk of late rectal adverse events. On the other hand, no dosimetric values were significantly associated with a risk of complication or disease progression (28). In consideration of these data, the preservation of the rectum, in the absence of target uncovering, is extremely important. The use of a rectal spacer through the application of an injectable and absorbable hydrogel designed to establish a transient separation between the rectum can be very useful. Mahal et al. demonstrated the feasibility of hydrogel spacer placement in PC patients previously treated with RT. Their study highlighted successful placement in 73% of cases, resulting in a statistically significant increase in the median distance between the prostate and the rectum post-brachytherapy. Despite challenges in some cases due to fibrosis and adhesions, the technique showed promising results in enhancing spatial separation and potentially reducing gastrointestinal and genitourinary toxicities (51). Uhl et al.’s prospective phase II study further substantiates these benefits, encompassing 52 men who underwent transperineal hydrogel injection followed by intensity-modulated RT. The study reported minimal acute gastrointestinal toxicity and no severe late toxicities at the 12-month follow-up, underscoring the hydrogel’s efficacy in maintaining rectal safety (52). Moreover, Patel et al.’s retrospective analysis underscored the acute benefits of a rectal spacer in combination with EBRT and LDR brachytherapy. Significant reductions in acute intestinal toxicities and rectal bleeding incidences within 3 months post-treatment were observed, reaffirming the spacer’s role in ameliorating treatment-related side effects (53).

Our case report aligns with these findings. This case with recurrent PC managed with salvage LDR brachytherapy and a rectal spacer demonstrated a significant reduction in PSA levels and the absence of urinary or gastrointestinal disturbances in the short term. The possibility of moving the rectum away from the prostate for an average distance of 5 mm made it possible to significantly reduce the dose received by the rectum while being able to deliver an ablative dose to the target.

Extreme attention must be paid to the hydrodissection step since in some cases, the fibrosis linked to the first RT treatment can be extremely difficult or even not feasible. Therefore, this procedure must be carried out by physicians with a high learning curve.

Secondly, it must always be taken into consideration that the implantation of the rectal spacer before positioning the iodine-125 seeds could induce the presence of artifacts in the ultrasound image, making the ultrasound-guided delineation of the prostate and the target more complicated. The presence of artifacts lengthens the time dedicated to contouring and therefore the total time of the procedure and the risk of treatment uncertainty, as demonstrated by **Figure 2**. In fact, the limit of the lobe of the prostate (in our case) is difficult to visualize. In this case, the expertise of the brachytherapy physician and the possibility of an ultrasound with axial and sagittal vision allowed the treatment to be completed. However, in some cases, the presence of artifacts could make visualization of the prostate and especially the urethra impossible.

Following this experience and the risk linked to the presence of an artifact, our center opted to implant the OAR spacer after the positioning of the radioactive seeds. The downside of this approach is that the rectal dose collected during the dosimetry will not be accurate in real life, since the spacer will be positioned after the dosimetry.

## 4 Conclusion

In our knowledge, this is the first paper reporting the use of a rectal spacer in a reirradiation setting via LDR brachytherapy. Our experience advocates for the integration of a hydrogel rectal spacer as a valuable tool in PC reirradiation protocols, offering a strategic approach to optimize treatment safety by minimizing rectal toxicity and enhancing patient-reported outcomes. Extreme attention must be paid to the hydrodissection step, and we think that it would be better to implant the rectal spacer after positioning the seeds because the risk of artifacts could make contouring difficult.

## Data Availability

The original contributions presented in the study are included in the article/supplementary material. Further inquiries can be directed to the corresponding authors.

## References

[1] Bray F Ferlay J Soerjomataram I Siegel RL Torre LA Jemal A. Global cancer statistics 2018: GLOBOCAN estimates of incidence and mortality worldwide for 36 cancers in 185 countries. CA Cancer J Clin. (2018) 68:394−424. doi: 10.3322/caac.21492 30207593

[2] Torre LA Siegel RL Ward EM Jemal A. Global cancer incidence and mortality rates and trends—An update. Cancer Epidemiol Biomarkers Prev. (2016) 25:16−27. doi: 10.1158/1055-9965.EPI-15-0578 26667886

[3] Cozzi S Ruggieri MP Alì E Ghersi SF Vigo F Augugliaro M. Moderately hypofractionated helical tomotherapy for prostate cancer: ten-year experience of a mono-institutional series of 415 patients. In Vivo. (2023) 37:777−85. doi: 10.21873/invivo.13141 36881094 PMC10026640

[4] Munoz F Fiorica F Caravatta L Rosa C Ferella L Boldrini L. Outcomes and toxicities of re-irradiation for prostate cancer: A systematic review on behalf of the Re-Irradiation Working Group of the Italian Association of Radiotherapy and Clinical Oncology (AIRO). Cancer Treat Rev. (2021) 95:102176. doi: 10.1016/j.ctrv.2021.102176 33743409

[5] Cozzi S Finocchi Ghersi S Bardoscia L Najafi M Blandino G Alì E. Linac-based stereotactic salvage reirradiation for intraprostatic prostate cancer recurrence: toxicity and outcomes. Strahlenther Onkol. (2023) 199:554−64. doi: 10.1007/s00066-023-02043-3 36732443 PMC10212806

[6] Montoya J Gross E Karsh L. How I Do It: Hydrogel spacer placement in men scheduled to undergo prostate radiotherapy. Can J Urol. (2018) 25:9288−93. 29680009

[7] Hamstra DA Mariados N Sylvester J Shah D Karsh L Hudes R. Continued benefit to rectal separation for prostate radiation therapy: final results of a phase III trial. Int J Radiat Oncol. (2017) 97:976−85. doi: 10.1016/j.ijrobp.2016.12.024 28209443

[8] Cozzi S Botti A Timon G Blandino G Najafi M Manicone M. Prognostic factors, efficacy, and toxicity of involved-node stereotactic body radiation therapy for lymph node oligorecurrent prostate cancer: An investigation of 117 pelvic lymph nodes. Strahlenther Onkol. (2022) 198:700−9. doi: 10.1007/s00066-021-01871-5 34757443

[9] Cozzi S Bardoscia L Najafi M Botti A Blandino G Augugliaro M. Adenoid cystic carcinoma/basal cell carcinoma of the prostate: overview and update on rare prostate cancer subtypes. Curr Oncol. (2022) 29:1866−76. doi: 10.3390/curroncol29030152 35323352 PMC8947681

[10] Bardoscia L Triggiani L Sandri M Francavilla S Borghetti P Dalla Volta A. Non-metastatic ductal adenocarcinoma of the prostate: pattern of care from an uro-oncology multidisciplinary group. World J Urol. (2021) 39:1161−70. doi: 10.1007/s00345-020-03315-8 32591899

[11] Baty M Créhange G Pasquier D Palard X Deleuze A Gnep K. Salvage reirradiation for local prostate cancer recurrence after radiation therapy. For who? When? How? Cancer/Radiothérapie. (2019) 23:541−58. doi: 10.1016/j.canrad.2019.07.125 31421999

[12] Nguyen PL D’Amico AV Lee AK Warren Suh W. Patient selection, cancer control, and complications after salvage local therapy for postradiation prostate-specific antigen failure: A systematic review of the literature. Cancer. (2007) 110:1417−28. doi: 10.1002/cncr.v110:7 17694553

[13] Paoletti L Ceccarelli C Menichelli C Aristei C Borghesi S Tucci E. Special stereotactic radiotherapy techniques: procedures and equipment for treatment simulation and dose delivery. Rep Pract Oncol Radiother. (2022) 27:1−9. doi: 10.5603/RPOR.a2021.0129 35402024 PMC8989452

[14] Bardoscia L Pasinetti N Triggiani L Cozzi S Sardaro A. Biological bases of immune-related adverse events and potential crosslinks with immunogenic effects of radiation. Front Pharmacol. (2021) 12:746853. doi: 10.3389/fphar.2021.746853 34790123 PMC8591245

[15] Cozzi S Alì E Bardoscia L Najafi M Botti A Blandino G. Stereotactic body radiation therapy (SBRT) for oligorecurrent/oligoprogressive mediastinal and hilar lymph node metastasis: A systematic review. Cancers. (2022) 14:2680. doi: 10.3390/cancers14112680 35681659 PMC9179886

[16] Iori F Bruni A Cozzi S Ciammella P Di Pressa F Boldrini L. Can radiotherapy empower the host immune system to counterattack neoplastic cells? A systematic review on tumor microenvironment radiomodulation. Curr Oncol. (2022) 29:4612−24. doi: 10.3390/curroncol29070366 35877226 PMC9319790

[17] Iori F Botti A Ciammella P Cozzi S Orlandi M Iori M. How a very large sarcomatoid lung cancer was efficiently managed with lattice radiation therapy: a case report. Ann Palliat Med. (2022) 11:3555−61. doi: 10.21037/apm-22-246 35871277

[18] Najafi M Jahanbakhshi A Gomar M Iotti C Giaccherini L Rezaie O. State of the art in combination immuno/radiotherapy for brain metastases: systematic review and meta-analysis. Curr Oncol. (2022) 29:2995−3012. doi: 10.3390/curroncol29050244 35621634 PMC9139474

[19] Navarria P Minniti G Clerici E Comito T Cozzi S Pinzi V. Brain metastases from primary colorectal cancer: is radiosurgery an effective treatment approach? Results of a multicenter study of the radiation and clinical oncology Italian association (AIRO). Br J Radiol. (2020) 93:20200951. doi: 10.1259/bjr.20200951 33035077 PMC7716018

[20] Cuccia F Mazzola R Nicosia L Giaj-Levra N Figlia V Ricchetti F. Prostate re-irradiation: current concerns and future perspectives. Expert Rev Anticancer Ther. (2020) 20:947−56. doi: 10.1080/14737140.2020.1822742 32909471

[21] Zerini D Jereczek-Fossa BA Ciabattoni A Mirri A Bertoni F Fersino S. PROLAPSE: survey about local prostate cancer relapse salvage treatment with external beam re-irradiation: results of the Italian Association of Radiotherapy and Clinical Oncology (AIRO). J Cancer Res Clin Oncol. (2020) 146:2311−7. doi: 10.1007/s00432-020-03297-5 32583236 PMC11804665

[22] Ingrosso G Becherini C Lancia A Caini S Ost P Francolini G. Nonsurgical salvage local therapies for radiorecurrent prostate cancer: A systematic review and meta-analysis. Eur Urol Oncol. (2020) 3:183−97. doi: 10.1016/j.euo.2018.12.011 31411996

[23] Baumann BC Baumann JC Christodouleas JP Soffen E. Salvage of locally recurrent prostate cancer after external beam radiation using reduced-dose brachytherapy with neoadjuvant plus adjuvant androgen deprivation. Brachytherapy. (2017) 16:291−8. doi: 10.1016/j.brachy.2016.12.011 28139422

[24] Mbeutcha A Chauveinc L Bondiau PY Chand ME Durand M Chevallier D. Salvage prostate re-irradiation using high-dose-rate brachytherapy or focal stereotactic body radiotherapy for local recurrence after definitive radiation therapy. Radiat Oncol. (2017) 12:49. doi: 10.1186/s13014-017-0789-9 28274241 PMC5343540

[25] Wong WW Buskirk SJ Schild SE Prussak KA Davis BJ. Combined prostate brachytherapy and short-term androgen deprivation therapy as salvage therapy for locally recurrent prostate cancer after external beam irradiation. J Urol. (2006) 176:2020−4. doi: 10.1016/j.juro.2006.07.008 17070243

[26] Nguyen PL Chen M D’Amico AV Tempany CM Steele GS Albert M. Magnetic resonance image-guided salvage brachytherapy after radiation in select men who initially presented with favorable-risk prostate cancer: A prospective phase 2 study. Cancer. (2007) 110:1485−92. doi: 10.1002/cncr.v110:7 17701957

[27] Lahmer G Lotter M Kreppner S Fietkau R Strnad V. Protocol-based image-guided salvage brachytherapy: Early results in patients with local failure of prostate cancer after radiation therapy. Strahlenther Onkol. (2013) 189:668−74. doi: 10.1007/s00066-013-0373-7 23824103

[28] Yamada Y Kollmeier MA Pei X Kan CC Cohen GN Donat SM. A Phase II study of salvage high-dose-rate brachytherapy for the treatment of locally recurrent prostate cancer after definitive external beam radiotherapy. Brachytherapy. (2014) 13:111−6. doi: 10.1016/j.brachy.2013.11.005 24373762 PMC5718052

[29] Jo Y Fujii T Hara R Yokoyama T Miyaji Y Yoden E. Salvage high-dose-rate brachytherapy for local prostate cancer recurrence after radiotherapy – preliminary results. BJU Int. (2012) 109:835−9. doi: 10.1111/j.1464-410X.2011.10519.x 21933327

[30] Łyczek J Kawczyńska MM Garmol D Kasprowicz A Kulik A Dąbkowski M. HDR brachytherapy as a solution in recurrences of locally advanced prostate cancer. J Contemp Brachyther. (2009) 1:105−8. PMC5075996 27795720

[31] Maenhout M Peters M Van Vulpen M Moerland MA Meijer RP Van Den Bosch MAAJ. Focal MRI-guided salvage high-dose-rate brachytherapy in patients with radiorecurrent prostate cancer. Technol Cancer Res Treat. (2017) 16:1194−201. doi: 10.1177/1533034617741797 29333958 PMC5762090

[32] Koutrouvelis P Hendricks F Lailas N Gil-Montero G Sehn J Khawand N. Salvage reimplantation in patient with local recurrent prostate carcinoma after brachytherapy with three dimensional computed tomography-guided permanent pararectal implant. Technol Cancer Res Treat. (2003) 2:339−44. doi: 10.1177/153303460300200409 12892517

[33] Burri RJ Stone NN Unger P Stock RG. Long-term outcome and toxicity of salvage brachytherapy for local failure after initial radiotherapy for prostate cancer. Int J Radiat Oncol. (2010) 77:1338−44. doi: 10.1016/j.ijrobp.2009.06.061 20138442

[34] Lacy JM Wilson WA Bole R Chen L Meigooni AS Rowland RG. Salvage brachytherapy for biochemically recurrent prostate cancer following primary brachytherapy. Prostate Cancer. (2016) 2016:1−9. doi: 10.1155/2016/9561494 PMC4820628 27092279

[35] Peters M Maenhout M van der Voort Van Zyp JRN Moerland MA Moman MR Steuten LMG. Focal salvage iodine-125 brachytherapy for prostate cancer recurrences after primary radiotherapy: A retrospective study regarding toxicity, biochemical outcome and quality of life. Radiother Oncol. (2014) 112:77−82. doi: 10.1016/j.radonc.2014.06.013 24998704

[36] Vargas C Swartz D Vashi A Blasser M Kasraeian A Cesaretti J. Salvage brachytherapy for recurrent prostate cancer. Brachytherapy. (2014) 13:53−8. doi: 10.1016/j.brachy.2013.10.012 24295965

[37] Allen GW Howard AR Jarrard DF Ritter MA. Management of prostate cancer recurrences after radiation therapy-brachytherapy as a salvage option. Cancer. (2007) 110:1405−16. doi: 10.1002/cncr.v110:7 17685384

[38] Lee B Shinohara K Weinberg V Gottschalk AR Pouliot J Roach M. Feasibility of high-dose-rate brachytherapy salvage for local prostate cancer recurrence after radiotherapy: The University of California–San Francisco experience. Int J Radiat Oncol. (2007) 67:1106−12. doi: 10.1016/j.ijrobp.2006.10.012 17197119

[39] Aaronson DS Yamasaki I Gottschalk A Speight J Roach M Shinohara K. Salvage permanent perineal radioactive seed implantation for the treatment of localized prostate adenocarcinoma recurrence after external beam radiation. Int J Radiat Oncol. (2008) 72:S310. doi: 10.1016/j.ijrobp.2008.06.1078 19245439

[40] Chen CP Weinberg V Shinohara K Roach M Nash M Gottschalk A. Salvage HDR brachytherapy for recurrent prostate cancer after previous definitive radiation therapy: 5-year outcomes. Int J Radiat Oncol. (2013) 86:324−9. doi: 10.1016/j.ijrobp.2013.01.027 23474112

[41] Shimbo M Inoue K Koike Y Katano S Kawashima K. Salvage ^125^I seed implantation for prostate cancer with postradiation local recurrence. Urol Int. (2013) 90:294−300. 23467122 10.1159/000346322

[42] Kukiełka AM Hetnał M Dąbrowski T Walasek T Brandys P Nahajowski D. Salvage prostate HDR brachytherapy combined with interstitial hyperthermia for local recurrence after radiation therapy failure. Strahlenther Onkol. (2014) 190:165−70. doi: 10.1007/s00066-013-0486-z 24317192

[43] Barbera F Triggiani L Buglione M Ghirardelli P Vitali P Caraffini B. Salvage low dose rate brachytherapy for recurrent prostate cancer after external beam radiotherapy: results from A single institution with focus on toxicity and functional outcomes. Clin Med Insights Oncol. (2017) 11:117955491773876. doi: 10.1177/1179554917738765 PMC5680931 29151782

[44] Moman MR van der Poel HG Battermann JJ Moerland MA Van Vulpen M. Treatment outcome and toxicity after salvage 125-I implantation for prostate cancer recurrences after primary 125-I implantation and external beam radiotherapy. Brachytherapy. (2010) 9:119−25. doi: 10.1016/j.brachy.2009.06.007 19850536

[45] Hsu CC Hsu H Pickett B Crehange G Hsu ICJ Dea R. Feasibility of MR imaging/MR spectroscopy-planned focal partial salvage permanent prostate implant (PPI) for localized recurrence after initial PPI for prostate cancer. Int J Radiat Oncol. (2013) 85:370−7. doi: 10.1016/j.ijrobp.2012.04.028 22672747

[46] Rose JN Crook JM Pickles T Keyes M Morris WJ. Salvage low-dose-rate permanent seed brachytherapy for locally recurrent prostate cancer: Association between dose and late toxicity. Brachytherapy. (2015) 14:342−9. doi: 10.1016/j.brachy.2015.01.002 25727178

[47] Wojcieszek P Szlag M Głowacki G Cholewka A Gawkowska-Suwińska M Kellas-Ślęczka S. Salvage high-dose-rate brachytherapy for locally recurrent prostate cancer after primary radiotherapy failure. Radiother Oncol. (2016) 119:405−10. doi: 10.1016/j.radonc.2016.04.032 27165612

[48] Cuccia F Nicosia L Mazzola R Figlia V Giaj-Levra N Ricchetti F. Linac-based SBRT as a feasible salvage option for local recurrences in previously irradiated prostate cancer. Strahlenther Onkol. (2020) 196:628−36. doi: 10.1007/s00066-020-01628-6 32399638

[49] Cuccia F Rigo M Figlia V Giaj-Levra N Mazzola R Nicosia L. 1.5T MR-guided daily adaptive stereotactic body radiotherapy for prostate re-irradiation: A preliminary report of toxicity and clinical outcomes. Front Oncol. (2022) 12:858740. doi: 10.3389/fonc.2022.858740 35494082 PMC9043550

[50] Dipasquale G Zilli T Fiorino C Rouzaud M Miralbell R. Salvage reirradiation for local failure of prostate cancer after curative radiation therapy: Association of rectal toxicity with dose distribution and normal-tissue complication probability models. Adv Radiat Oncol. (2018) 3:673−81. doi: 10.1016/j.adro.2018.06.001 30370369 PMC6200893

[51] Mahal BA Ziehr DR Hyatt AS Neubauer-Sugar EH O’Farrell DA O’Leary MP. Use of a rectal spacer with low-dose-rate brachytherapy for treatment of prostate cancer in previously irradiated patients: Initial experience and short-term results. Brachytherapy. (2014) 13:442−9. doi: 10.1016/j.brachy.2014.05.001 24880584

[52] Uhl M Van Triest B Eble MJ Weber DC Herfarth K De Weese TL. Low rectal toxicity after dose escalated IMRT treatment of prostate cancer using an absorbable hydrogel for increasing and maintaining space between the rectum and prostate: Results of a multi-institutional phase II trial. Radiother Oncol. (2013) 106:215−9. doi: 10.1016/j.radonc.2012.11.009 23333011

[53] Patel AK Houser C Benoit R Smith RP Beriwal S. Acute patient-reported bowel quality of life and rectal bleeding with the combination of prostate external beam radiation, low-dose-rate brachytherapy boost, and SpaceOAR. Brachytherapy. (2020) 19:477−83. doi: 10.1016/j.brachy.2020.03.006 32331976

